# Assessing the Effectiveness of A-PRF+ for Treating Periodontal Defects: A Prospective Interventional Pilot Study Involving Smokers

**DOI:** 10.3390/medicina60111897

**Published:** 2024-11-19

**Authors:** Ada Stefanescu, Dorin Ioan Cocoș, Gabi Topor, Fabian Cezar Lupu, Doriana Forna-Agop, Kamel Earar

**Affiliations:** 1 Faculty of Dental Medicine, “Dunarea de Jos” University, 800008 Galati, Romania; ada.stefanescu@ugal.ro (A.S.); gabi.topor@ugal.ro (G.T.); kamel.earar@ugal.ro (K.E.); 2 Mechanical Engineering, Mechatronics and Robotics Department, “Gheorghe Asachi” Technical University of Iasi, 700050 Iasi, Romania; 3 Department of Maxillo-Facial Surgery, “Grigore T. Popa” University of Medicine and Pharmacy, 700050 Iasi, Romania; doriana.agop-forna@umfiasi.ro

**Keywords:** clinical attachment loss, A-PRF+, periodontitis, smokers, non-smokers, probing depth

## Abstract

*Background and Objectives:* This study aimed to evaluate the effects of advanced platelet-rich fibrin (A-PRF+) tissue regeneration therapy on clinical periodontal parameters in non-smokers and smoker patients. The anticipated biological mechanisms of A-PRF+ include stimulating angiogenesis, thereby promoting the formation of new blood vessels, which is essential for tissue healing. Additionally, A-PRF+ harnesses the regenerative properties of platelet-rich fibrin, contributing to the repair and regeneration of periodontal tissues. *Materials and Methods:* The study was conducted on 55 patients, divided into two groups: non-smoker patients (n = 29) and smoker patients (n = 26). A single operator conducted the surgical procedure. Following the administration of local anesthesia with articaine 4% with adrenaline 1:100,000 precise intracrevicular incisions were made, extending towards the adjacent teeth, until the interproximal spaces, with meticulous attention to conserving the interdental gingival tissue to the greatest extent possible. Extended, full-thickness vestibular and oral flaps were carefully lifted, and all granulation tissue was meticulously removed from the defect without altering the bone contour. After debridement of the defects, A-PRF+ was applied. BOP (bleeding on probing), PI (plaque index), CAL (clinical attachment loss), and probing depth (PD) were determined at baseline and six months post-surgery. *Results:* Significant reductions were observed in PD and CAL after six months. In the non-smokers group, PD decreased from 7.0 ± 1.0 mm to 3.1 ± 0.1 mm (*p* < 0.001), while in the smokers group, PD decreased from 6.9 ± 1.1 mm to 3.9 ± 0.3 mm (*p* < 0.001). CAL decreased in the non-smokers group from 5.8 ± 0.7 mm to 2.6 ± 0.2 mm and from 5.7 ± 0.9 mm to 3.2 ± 0.2 mm (*p* < 0.001) in smokers. Notably, the reductions in CAL and PD were statistically more significant in the non-smokers group. *Conclusions:* Even though the clinical periodontal improvements were considerable in smoker patients, they did not reach the level observed in non-smoker patients.

## 1. Introduction

Maintaining optimal periodontal health is essential for overall well-being, directly impacting oral hygiene and systemic health. However, achieving this can be particularly challenging, especially among individuals who smoke, as smoking is widely recognized as a significant risk factor for compromised oral health [1,2]. In light of these challenges, there is a growing interest in exploring novel and effective interventions to address perio-dontal issues.

One such promising therapeutic modality that has garnered attention is advanced platelet-rich fibrin (A-PRF+). This innovative approach utilizes the regenerative proper-ties of platelet-rich fibrin to promote tissue healing and regeneration in the periodontal environment [3,4]. By harnessing the body’s natural healing mechanisms, A-PRF+ offers potential benefits for improving periodontal health and addressing the specific challenges of smoking [5,6].

Smoking is widely recognized for its adverse effects on the periodontium, presenting a significant obstacle to achieving successful outcomes with traditional periodontal treatments [5]. In the quest to overcome these challenges and enhance periodontal therapy outcomes, researchers have turned their attention to A-PRF+. This innovative approach harnesses the regenerative potential of concentrated growth factors and cytokines within platelet-rich fibrin to promote tissue healing and regeneration in periodontal defects [4,7].

The current study aimed to assess the periodontal clinical effects of platelet-rich fibrin (A-PRF+) tissue regeneration therapy in both smoking and non-smokers patients. Platelet concentrates have found widespread application in both medical and dental fields due to their rapid stimulation of angiogenesis and potential for tissue regeneration [3,8]. Platelet-rich fibrin (PRF) represents a second-generation platelet concentrate designed to mitigate contamination risks by eliminating the need for anticoagulants or animal-derived thrombin. Initially described by Choukroun et al. as platelet-leukocyte-rich fibrin (L-PRF) [9], its production involves the collection of autologous blood from the patient followed by immediate centrifugation to induce platelet activation and fibrin polymerization. The centrifugation process forms a complex three-dimensional scaffold rich in various essential cellular components such as platelets, growth factors (including VEGF, PDGF, TGF-β1, EGF, IGF-I, and HGF), leukocytes, and cytokines, all of which are recognized for their piv-otal role in the process of regeneration and wound healing [3,10].

The use of advanced platelet-rich fibrin plus (A-PRF+) represents a cutting-edge approach in periodontal regeneration. However, its clinical efficacy specifically in smokers—a group known for challenging healing dynamics—remains underexplored.

Our research aims to provide new insights that could inform evidence-based protocols and enhance the effectiveness of periodontal regeneration therapies by examining the clinical outcomes of A-PRF+ in this high-risk population, aiming to explore and develop tailored treatments for individuals facing the challenges of smoking-induced periodontal defects.

## 2. Materials and Methods

### 2.1. Study Population

The study was carried out on a group of 55 patients, divided into two groups: smoker patients (n = 26) and non-smoker patients (n = 29). While the sample sizes of the two groups were not equal, statistical analyses were performed to account for this difference, and the results did not indicate a significant impact on the overall findings. Patients considered for surgical therapy followed the initial phase of periodontal treatment, which included scaling and root planing to reduce inflammation and bacterial load. Following this preparatory phase, open curettage was performed as the primary surgical intervention. This method allowed for direct visualization and removal of the remaining subgingival deposits, enhancing root surface debridement and access to periodontal defects. Patients were enrolled consecutively when the following inclusion criteria were met: (1) no systemic disease, such as diabetes and osteoporosis, conditions that could affect the outcome of therapy, without systemic medication or treatment; (2) a good level of oral hygiene with plaque index (PI) <20% and bleeding index on probing (BOP) <20%; (3) the presence of a minimum of one or more intrabony defects of 2 or 3 walls, combined with a defect angle of 20–40 (±5) degrees, since the angle of the radiographic defect influences the outcome of regenerative surgical therapy in the intrabony defects and with a minimum probing depth (PD) of 6 mm and intrabony component of at least 4 mm.

Patients were informed about the study protocol, and signed informed consent was obtained from each subject. The study was carried out according to the rules of the Decla-ration of Helsinki and was approved by the Ethics Committee of “Dunarea de Jos” Uni-versity, Galati, Romania (no. 242/CEU).

### 2.2. Clinical Examination

All patients underwent a comprehensive clinical examination by a single clinical examiner, establishing the following periodontal parameters: plaque index (PI), bleeding on probing (BOP), probing depth (PD), and clinical periodontal attachment loss (CAL).

To calibrate the clinical examination, we used five patients, each presenting ten teeth (mono-radicular and multi-radicular) with PD > 6 mm on at least one surface of each tooth. The examiner assessed the patients on two separate occasions, 48 h apart. Calibration was accepted if >90% of the recordings could be reproduced with a difference of 1.0 mm.

Clinical periodontal examination was performed at baseline, seven days before surgical therapy, and six months after. The WHO periodontal probe was used, and each tooth, both mono- and multi-radicular, was measured at three points on each surface.

Although standardized probing devices, such as the “Florida Probe”, were not used in this study, the examiner was carefully trained and calibrated to minimize variability between measurements.

The primary outcome variables under consideration were changes in probing depth and clinical attachment loss.

### 2.3. Preparation of A-PRF+

Just before the surgery, all patients underwent preparation of A-PRF+ using a commercially available PRF kit alongside the “Process for PRF Duo” centrifuge (Chouk-roun). Ulnar venous blood was collected from the patient into two 10 mL vacuum tubes (labeled as A-PRF+ tubes) without adding an anticoagulant. Subsequently, the blood samples were immediately centrifuged at 1300 rpm for 8 min, followed by a resting period of 5 min.

**Figure 1:**
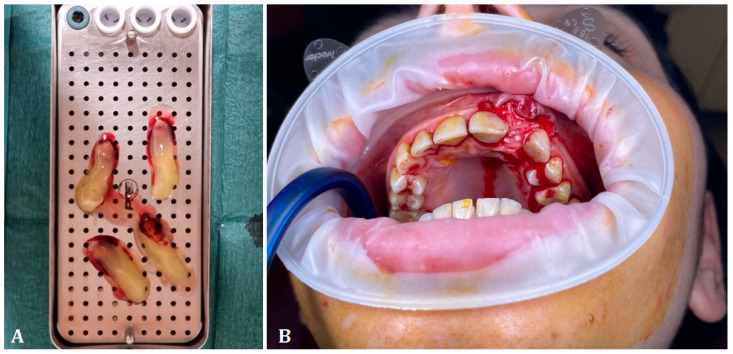
Processing and application of A-PRF+. (**A**) The A-PRF+ clots are prepared to be inserted intraoperatively. (**B**) Placement of A-PRF+ clots at the level of intra-osseous defects (distal at 2.1 and mesial at 2.3).

During the centrifugation process, the blood within the tubes underwent layering, representing the initial stages of coagulation. The uppermost layer comprised platelet-poor plasma (PPP), the middle layer consisted of platelet-rich fibrin clot (PRF), and the bottom layer contained red blood cells (RBCs). The PRF clot, which remained gel-like, was carefully separated from the tube, rid of red blood cells, and utilized as a gel for the procedure (Figure 1).

### 2.4. Surgical Procedure

A single operator conducted the surgical procedure. Following the administration of local anesthesia, using articaine 4% with adrenaline 1:100,000, precise intracrevicular incisions were made, extending towards the adjacent teeth, until the interproximal spaces, with meticulous attention to conserving the interdental gingival tissue to the greatest extent possible. Extended, full-thickness vestibular and oral flaps were carefully lifted, and all granulation tissue was meticulously removed from the defect without altering the bone contour.

A Gracey Mini Five from HuFriedy was used to perform the root planing. After the defects were debrided, A-PRF+ was applied. Finally, the flap was repositioned to its original placement and securely closed using either vertical or horizontal sutures with 5.0 thread (Biotex 5.0, Biotex Inc, Houston, TX, USA).

### 2.5. Postoperative Care

Each patient was prescribed antibiotics for one week, to be taken twice daily (Aug-mentin Duo, comprising 875 mg of amoxicillin and 125 mg of clavulanic acid, manufac-tured by GlaxoSmithKline, Brentford, UK). Postoperative care involved rinsing with 0.2% chlorhexidine (Curasept ADS 220, manufactured by Curaden AG, Kriens, Switzerland) twice daily for two weeks. Sutures were removed two weeks following the surgery.

Detailed instructions were provided to ensure proper oral hygiene maintenance. Study participants were scheduled for follow-up visits every week during the first month after the surgery, followed by an appointment at six months.

### 2.6. Statistical Analysis

A descriptive analysis was employed in analyzing clinical parameters, presenting results as mean ± SD and range at baseline and the 6-month interval. The normality assessment was conducted using the Shapiro–Wilk test. Changes within the groups were evaluated using paired t-tests, with *p* values < 0.05 deemed statistically significant.

We determined the group sizes using a probing depth (PD) change of 1 mm as the main variable, with a standard deviation of 0.2, a power of 90%, and an alpha set to 0.05. This led to 23 subjects per group. To account for potential dropouts from the study, we set the group size at 26 patients per group, considering an estimated dropout rate of 10%. The software we used is IBM SPSS Statistics V22.0.

## 3. Results

In the present study, 55 patients divided into two groups were enrolled: smoker pa-tients (F) (n = 26) and non-smoker patients (N) (n = 29). The mean age was 42.4 ± 2.7 years (group F) and 41.7 ± 4.6 years (group N), respectively, with no statistically significant difference (*p* = 0.753). Demographic data for both groups are shown in Table 1.

At the baseline, we did not notice statistically significant differences between the groups regarding the investigated clinical parameters.

The PI followed a slight increase at six months for both the smokers group (from 12.5 ± 1.0 to 13.9 ± 2.3) and the non-smokers group (from 11.8 ± 1.9 to 14.2 ± 3.1), without reaching the level of statistical significance (Table 2).

The same trend was seen for the BOP; it increased from 9.6 ± 1.7 to 11.8 ± 2.0 for smoker subjects and from 10.0 ± 1.7 to 12.2 ± 2.1 for non-smoker subjects, still statistically insignificant differences (Table 2).

However, significant reductions were observed for PD and CAL at six months. In the smokers group, PD decreased from 6.9 ± 1.1 mm to 3.9 ± 0.3 mm (*p* < 0.001), and in the non-smokers group, it decreased from 7.0 ± 1.0 mm to 3.1 ± 0.1 mm (*p* < 0.001) (Table 2).

CAL decreased in the smokers group from 5.7 ± 0.9 mm to 3.2 ± 0.2 mm (*p* < 0.001) and from 5.8 ± 0.7 mm to 2.6 ± 0.2 mm in the non-smokers. It is worth noting, however, that the decreases in PD and CAL were more statistically significant for the non-smokers group (Table 2).

## 4. Discussion

Platelet-rich fibrin (PRF) serves as a matrix within fibrin that facilitates angiogenesis, cellular proliferation, differentiation, and chemotaxis, effectively functioning as a biomi-metic reservoir for both cells and cell signaling processes [11]. Consequently, its applications extend to various realms such as periodontal regeneration (including gingival recession treatment), alveolar ridge preservation, sinus lifting procedures, management of skin ulcers/necrosis, chronic wound healing, plastic and reconstructive surgery, as well as addressing musculoskeletal and tendon injuries [12].

To maintain the integrity of growth factors (GF) within leukocytes and platelets, a reduction in rotational centrifugal force (RCF) is necessary. High RCF levels decrease cell count and adversely impact the release of growth factors [13].

Platelets undergo recovery from the effects of centrifugal force during periods of rest. Nonetheless, the larger, metabolically active platelets with heightened prothrombotic po-tential are eliminated through high-speed centrifugation, as corroborated by light trans-mission aggregometry (LTA) analysis findings, resulting in a potential decrease in aggre-gation [14]. Notably, TGF-β remains unaffected by G forces [15].

Blood coagulation initiates swiftly, even before the tubes are subjected to centrifuga-tion. Plastic tubes containing siliceous substances promote this activation of coagulation, necessitating a recommended time frame of 60–90 s per every five collection tubes between blood withdrawal and the commencement of centrifugation. Failure to adhere to this timeframe can significantly impact the size of the PRF membrane, causing notable reduction [16]. Simultaneously, TGF-β is released upon platelet activation, binding to the newly formed fibrin-rich extracellular matrix and shielded from centrifugal forces [15].

In recent years, modifications to the original L-PRF protocol, such as adjustments to spin time and G-force, have given rise to A-PRF, a technique also pioneered by Chouk-roun. A-PRF involves a reduction in rotational speed during centrifugation and an extension of time [17]. This advancement signifies enhanced mechanical and biological properties compared to L-PRF, resulting in improved outcomes. Immunohistochemical studies have revealed a distinct cellular distribution pattern with A-PRF when subjected to low G forces. There is a notable dispersion of platelets and leukocytes throughout the clot, along with an intriguing increase in granulocytic neutrophil populations in the distal portion of the collected supernatant, a phenomenon not observed following high centrifugation forces [18].

Drawing from the published scientific literature, the reticular and porous microstructure of A-PRF is credited with its elasticity, which facilitates the encapsulation of various cellular components within the interfibrillar spaces of the membrane, a phenomenon already substantiated [19]. This inherent property enables increased migration, fibroblast proliferation, and elevated collagen mRNA levels [20].

Fuijoka-Kobayashi et al. [21] proposed a novel product, termed A-PRF+, which re-duced the rotation time while maintaining the same centrifugal force applied in the A-PRF protocol. Despite a slight decrease in duration, A-PRF+ is believed to offer distinct ad-vantages, notably in preserving cells within the formed clot, consequently leading to fur-ther enhancements in its characteristics compared to the A-PRF mentioned above.

Evidence has already substantiated the highest percentage of cells that can be col-lected, along with greater uniformity in platelet distribution and porosity compared to A-PRF. This phenomenon is crucial for balancing ensnared cells and essential processes such as chemotaxis, migration, proliferation, and degradability, all closely tied to the sus-tained release of growth factors [22]. The authors confirmed the hypothesis that alterations in the mechanical properties of A-PRF and A-PRF+ could lead to variations in the release of specific groups of growth factors. For instance, during a limited measurement period, significantly elevated levels of PDGF and TGF-β1 were observed within the scaffold on days 7 and 10. Regarding EGF, peak release occurred early in the 24 h and was more pronounced in A-PRF+. Notably, VEGF accumulation was particularly notable in A-PRF+, possibly attributed to its affinity with the fibrin and fibrinogen quantities within the orga-nized network [20].

The large surface cell collector (LSCC) employed in A-PRF and A-PRF+ creates larger diameter pores within the fibrin network. This facilitates the perfusion of cells and a few vessels to the peripheral edges of the scaffold [23].

One of the primary advantages of PRF lies in its fibrin network, which fosters the formation of blood clots and mechanisms for tissue repair [24]. Compared to PRP, the re-lease kinetics of growth factors in PRF appear to be slower, thereby influencing regenera-tion over a more prolonged duration [25]. An increasing number of studies are shedding light on the beneficial effects of leukocytes on healing, tissue regeneration, and, notably, the quality of the fibrin network. The presence of leukocytes within PRF contributes to its an-ti-infective and immunoregulatory functions [26], producing substantial quantities of VEGF. Alongside platelet-derived angiogenesis-stimulating factors, these elements may positively influence the provision of adequate blood supply to the healing site. Moreover, white blood cells play a role in the early stimulation of osteoprogenitor cells and facilitate the differentiation of monocytes into macrophages [17].

Numerous randomized controlled clinical trials have investigated the use of PRF for repairing and regenerating periodontal intrabony defects [27]. Across these studies, it was consistently demonstrated that the adjunctive application of PRF led to notable improve-ments in probing depth reductions and clinical attachment level gains compared to access flap debridement alone.

Incorporation of PRF with EMD did not yield any discernible differences between the study and control groups (EMD only) [28]. A clinical trial alongside a cone beam com-puted tomography study compared the effectiveness of PRF and EMD in treating intra-bony defects. Both materials effectively addressed such defects, although EMD exhibited significantly superior percent defect resolution [29]. Despite the statistically significant attachment gains and probing depth reductions observed with PRF in these clinical studies, it is imperative to underscore the necessity of histological examination to ascertain whether the outcomes align with periodontal regeneration or periodontal repair pro-cesses.

The biological advantages of PRF have been demonstrated to exert local effects by swiftly stimulating various cell types, influencing their recruitment, proliferation, and dif-ferentiation. According to existing literature, PRF prefers soft tissue regeneration over hard tissue [30]. In treating intrabony defects where space maintenance poses no issue, the formation of a blood clot alone may suffice; however, the supplementary use of PRF pri-marily serves as a scaffold and can enhance tissue regeneration when introduced into the periodontal pocket [31,32]. Further investigation is warranted to ascertain which compo-nents within PRF clots (such as cells/leukocytes, growth factors, or fibrin matrix) are crucial for expediting periodontal tissue regeneration.

Refinements to the preparation protocol, achieved by reducing the applied centrifu-gation force (RCF), have led to an enhanced preparation method for advanced PRF (A-PRF), utilizing an RCF of 208 g. Compared to PRF, the clot formed with A-PRF+ exhibi-ted a more porous structure with larger interfibrous spaces, facilitating uniform distribu-tion of cells, particularly platelets, throughout the clot. Furthermore, histological analysis of A-PRF+ revealed a significantly higher abundance of neutrophil granulocytes [30].

Fujioka-Kobayashi and colleagues (2017) discovered and documented that reducing both centrifugation speed and duration could enhance the total release of growth factors. A-PRF+ exhibited comparable porosity to A-PRF, with platelets evenly dispersed throughout the clot. These findings underscore the augmented regenerative potential of advanced PRF matrices [21].

The findings derived from this study, assessed at the six-month mark post-surgery, reveal noteworthy enhancements in probing depth and attachment loss across both groups. Notably, no adverse reactions were documented during the initial six-month pe-riod, suggesting a favorable tolerance of the autologous test material. However, it is perti-nent to highlight that the observed improvements were comparatively lower in smoker patients when juxtaposed with their non-smoker counterparts.

The detrimental impacts of smoking on the healing process are extensively docu-mented [33,34]. Studies in periodontal surgery, encompassing procedures such as dental implant placement, have reported compromised healing outcomes among smokers [35]. Nicotine, a primary constituent of tobacco, is swiftly absorbed through the buccal mucosa via diffusion, giving rise to various systemic effects [36]. Evidence suggests that nicotine hampers fibroblast functionality by impeding fibronectin activities and collagen synthesis while concurrently elevating collagenase activity, thereby promoting collagen degradation [37].

In individuals with periodontal disease, smokers exhibit diminished gingival bleed-ing compared to non-smokers, primarily due to the vasoconstrictive effects of nicotine, which reduce gingival blood flow [38]. This decrease in gingival vascularity impairs wound healing potential and heightens susceptibility to bacterial infections. Furthermore, research by Imamura and colleagues [39] elucidated that nicotine impedes the migration of epithelial cells essential for re-epithelialization during the wound healing process, act-ing through the MAPL ERK1/2 and p38 signaling pathways. Consequently, smoking is implicated in delayed wound healing and introduces a spectrum of complications, in-cluding infection, tissue necrosis, and epidermolysis.

Oxidative stress refers to the cellular damage caused by molecules that acquire oxy-gen from the body’s cells. Free radicals, also known as reactive oxygen species (ROS), are the most recognized agents capable of altering cellular DNA [40]. Oxidative stress manifests as an accumulation of oxidants within tissues, recognizing that oxidation is a natural physiological process. Consequently, oxidative stress contributes to premature cell aging and may be linked to various diseases, including cancer.

Numerous factors contribute to the generation of free radicals. These include expo-sure to a polluted environment, smoking, an imbalanced diet, sun exposure, stress, and intense physical exertion. Excessive production of antioxidants is also observed during periods of heightened anxiety, diabetes, obesity, cancer, and chronic inflammation. Conversely, insufficient antioxidant production may result from diabetes, smoking, or high blood pressure. Whether in the gums or bones, excessive soft tissue tension or bone pressure can lead to ischemia [40,41]. For instance, in smokers, the presence of smoke can deplete antioxidants in oral tissues (including bones and gingival tissue), skin, and lungs.

Instances of heightened or inadequate antioxidant production often coincide with a heightened risk of failures and complications. The healing process can be impeded during oxidative stress, potentially resulting in healing failures [42,43]. Hence, an intervention is often warranted: using PRF to mitigate this phenomenon.

The comparison of our findings with those of Csifó-Nagy et al. (2021) highlights critical differences in periodontal healing outcomes influenced by patient-specific factors, particularly smoking. While Csifó-Nagy et al. demonstrated comparable clinical efficacy between A-PRF+ and enamel matrix derivative (EMD) in a non-smoking population, our study revealed that smoking markedly impairs the regenerative potential of A-PRF+. This underscores the significant impact of smoking on periodontal tissue healing, manifested as reduced probing depth reduction and clinical attachment-level gains compared to non-smokers. The differences between our outcomes and those reported by Csifó-Nagy et al. emphasize the necessity of considering smoking status in regenerative periodontal therapy and highlight the need for further research into optimizing treatment strategies for smokers. Including this reference not only contextualizes our findings but also strengthens the understanding of how smoking alters the biological processes underpinning periodontal regeneration [44].

PRF, associated with oxidative stress, elicits three crucial actions: it promotes angio-genesis, exerts an anti-inflammatory effect, and impedes osteoclastogenesis. Consequently, PRF serves as a potent antioxidant. Notably, its activity span typically ranges from 7 to 15 days. However, in the context of the clinical study under scrutiny, more than these attributes were required to counteract the detrimental impact of smoking on tissue regeneration capacity when compared to non-smoker individuals.

The current study is not without its limitations. This is a clinical study. Future investigations are needed, such as radiologic and radio-imaging (CT, CBCT) for longer periods of time (6 months, 1 year, and 2 years) to demonstrate the stability of the results over time. First, there is a need for investigations involving larger cohorts to explore the beneficial effects of A-PRF+ on various subjects thoroughly. Expanding the study population would provide a more comprehensive understanding of the therapy’s efficacy and potential applications. Second, further research is warranted to delve into the molecular-level impact of this therapy, specifically on smokers. Understanding how A-PRF+ interacts with the biological mechanisms altered by smoking could shed light on its effectiveness in this subgroup of patients and potentially uncover novel therapeutic avenues. Therefore, while the present study provides valuable insights, future investigations addressing these limitations will contribute to a more nuanced understanding of A-PRF+ therapy.

## 5. Conclusions

A-PRF+ therapy stimulates angiogenesis and promotes the formation of new blood vessels, which are essential for tissue healing. Additionally, A-PRF+ contains growth factors that support periodontal regeneration and the repair of intraosseous defects, particularly in patients with smoking-induced deficiencies. The use of A-PRF+ led to significant improvements in periodontal parameters, such as the reduction of probing depth (PD) and clinical attachment loss (CAL) in both smokers and non-smokers, with better results observed in the non-smoking group. While smoker patients showed notable improvements, the beneficial effects of A-PRF+ were reduced compared to non-smokers. This highlights the negative impact of smoking on the healing process and periodontal regeneration capacity.

Utilizing a novel generation of PRF in the present study may open the way for ex-ploring the potential effects of platelet concentrates on periodontal healing. However, to solidify the findings of this investigation, further randomized clinical trials encompassing a more extensive and diverse population are warranted. Moreover, histological assess-ments focusing on periodontal regeneration will be imperative in corroborating the out-comes observed in this study.

Further clinical and imaging studies (CT, CBCT) over longer periods are needed to verify the stability of results and to explore the molecular interactions of A-PRF+ in smokers. Expanding this research could contribute to a more detailed understanding and optimization of therapeutic protocols for smoker patients.

## Figures and Tables

**Table 1:** Demographic data for study subjects.

Parameter		Group F (n = 26)	Group N (n = 29)
Age (years)		42.4 ± 2.7	41.7 ± 4.6
Gender	Male	14 (53.8%)	13 (44.8%)
	Female	12 (46.2%)	16 (55.2%)
Environment	Urban	15 (57.7%)	17 (58.6%)
	Rural	11 (42.3%)	12 (41.4%)

Group F: smokers. Group N: non-smoker subjects. Age is expressed as mean ± standard deviation; gender and background are expressed as a number (percentage).

**Table 2:** Changes in clinical parameters at baseline and after 6 months.

Parameter	Smokers (n = 26)			Non-Smokers (n = 29)			*p*-Value Intra-Group Baseline	*p*-Value Intra-Group After 6 Months
	Baseline	After 6 Months	*p*-Value Intra-Group	Baseline	After 6 Months	*p*-Value Intra-Group		
PI	12.54 ± 1.06	13.91 ± 2.33	0.064	11.82 ± 1.91	14.21 ± 3.15	0.059	0.199	0.168
BOP	9.62 ± 1.72	11.79 ± 1.95	0.057	10.01 ± 1.73	12.17 ± 2.13	0.055	0.108	0.423
PD (mm)	6.94 ± 1.12	3.89 ± 0.34 ^a^	<0.001	7.02 ± 1.08	3.12 ± 0.14 ^a,b^	<0.001	0.429	<0.001
CAL (mm)	5.68 ± 0.89	3.18 ± 0.18 ^a^	<0.001	5.81 ± 0.73	2.63 ± 0.21 ^a,b^	<0.001	0.961	<0.001

PI: plaque index. BOP: bleeding index on probing. PD: probing dept.; CAL: clinical periodontal attachment loss. Values are expressed as mean ± standard deviation. ^a^ indicates *p* < 0.05 in the same group after 6 months. ^b^ indicates *p* < 0.05 between groups at the same time point of assessment.

## Data Availability

Datasets are available from the authors upon reasonable request.
